# Calculation of virtual 3D subtraction angiographies using conditional generative adversarial networks (cGANs)

**DOI:** 10.1186/s12880-024-01454-7

**Published:** 2024-10-15

**Authors:** Sebastian Johannes Müller, Eric Einspänner, Stefan Klebingat, Seraphine Zubel, Roland Schwab, Erelle Fuchs, Elie Diamandis, Eya Khadhraoui, Daniel Behme

**Affiliations:** 1 Clinic for Neuroradiology, Otto-Von-Guericke-University Magdeburg, Leipziger Str. 44, D-39120 Magdeburg, Germany; 2 German Center for Neurodegenerative Diseases (DZNE), Leipziger Str. 44, D-39120 Magdeburg, Germany; 3 Stimulate Research Campus Magdeburg, Otto-Hahn-Str. 2, D-39106 Magdeburg, Germany

**Keywords:** *3D rotational angiography*, *Subtraction angiography*, pix2pix, Conditional generative adversarial network

## Abstract

**Objective:**

Subtraction angiographies are calculated using a native and a contrast-enhanced 3D angiography images. This minimizes both bone and metal artifacts and results in a pure image of the vessels. However, carrying out the examination twice means double the radiation dose for the patient. With the help of generative AI, it could be possible to simulate subtraction angiographies from contrast-enhanced 3D angiographies and thus reduce the need for another dose of radiation without a cutback in quality. We implemented this concept by using conditional generative adversarial networks.

**Methods:**

We selected all 3D subtraction angiographies from our PACS system, which had performed between 01/01/2018 and 12/31/2022 and randomly divided them into training, validation, and test sets (66%:17%:17%). We adapted the pix2pix framework to work on 3D data and trained a conditional generative adversarial network with 621 data sets. Additionally, we used 158 data sets for validation and 164 for testing. We evaluated two test sets with (*n* = 72) and without artifacts (*n* = 92). Five (blinded) neuroradiologists compared these datasets with the original subtraction dataset. They assessed similarity, subjective image quality, and severity of artifacts.

**Results:**

Image quality and subjective diagnostic accuracy of the virtual subtraction angiographies revealed no significant differences compared to the original 3D angiographies. While bone and movement artifact level were reduced, artifact level caused by metal implants differed from case to case between both angiographies without one group being significant superior to the other.

**Conclusion:**

Conditional generative adversarial networks can be used to simulate subtraction angiographies in clinical practice, however, new artifacts can also appear as a result of this technology.

## Introduction

3D angiographies are the gold standard for the detailed visualization of cerebral vessel and aneurysms located at the skull base [1, 2]. The classical use of subtraction mechanisms (3D subtraction angiographies, 3D DSA) may improve the image quality by reducing artifacts caused by arteriosclerotic plaques, bone structures or implants. To do this, the 3D rotation has to be performed twice, native and contrast-enhanced, which results in a double dose of radiation [3]. A combination of vascular pathologies, brain, and skull are seen on the contrast-enhanced series, therefore the value of the non-subtracted recordings are also recognized [4]. This raises the question as to whether performing a native rotation is really justified, or whether it is sufficient to create a simulated subtraction angiography using generative AI.

In 2014, conditional generative adversarial networks (cGANs) were introduced for training generative models [5]. These neuronal networks can create virtual 2D or 3D series from existing images (with or without contrast enhancement).

**Fig. 1:**
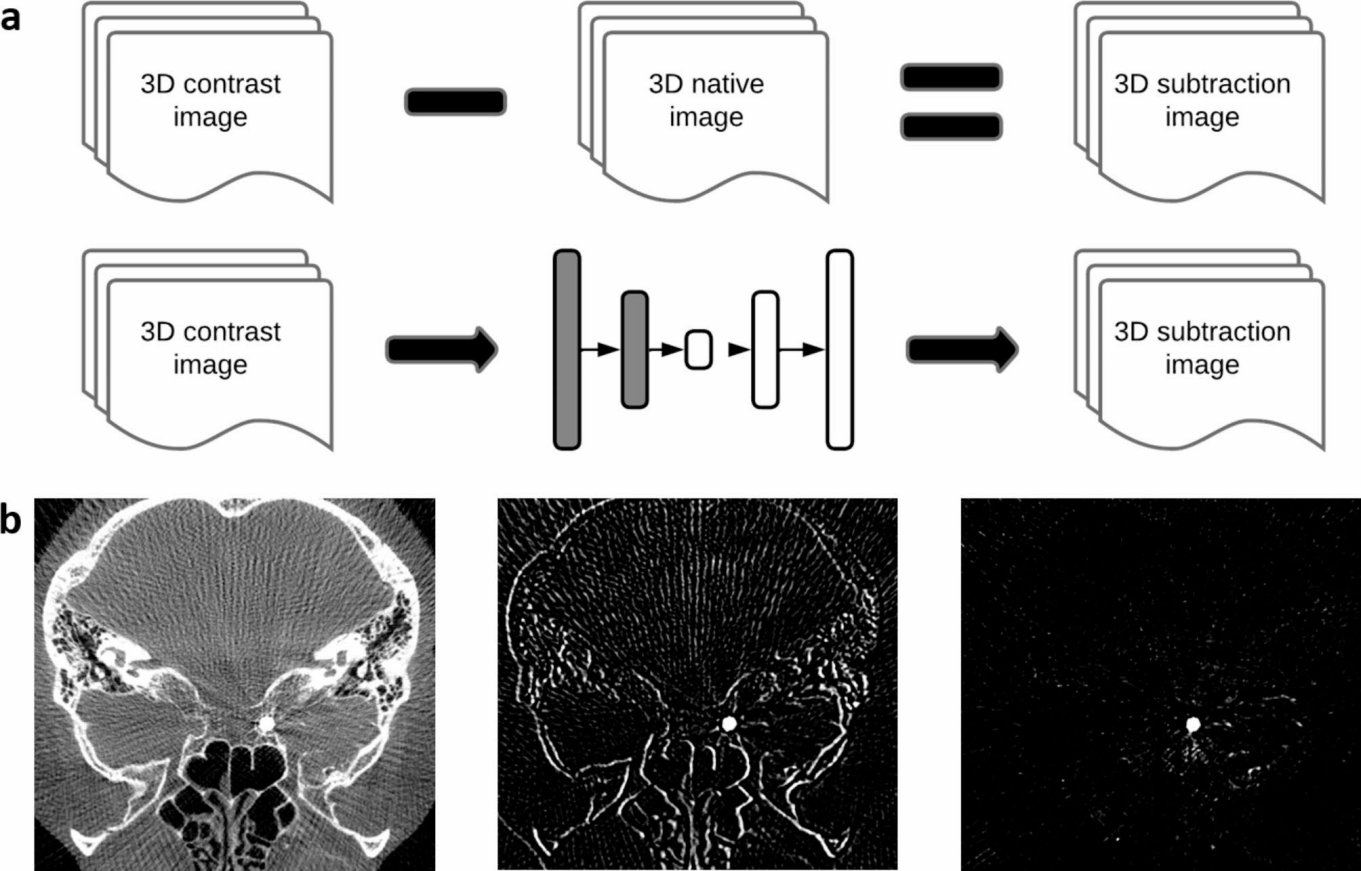
Schematic overview. **(a)** Basic concepts of subtraction imaging (top); and post-processing with neuronal networks / cGAN (middle); **(b)** 3D contrast-enhanced sequences (left), subtraction imaging with motion artifacts (middle) and subsequent generated GAN images (right);

We tried to simulate a rotational 3D DSA from the contrast-enhanced rotation angiographies using this approach. Finally, we compared the virtual 3D DSA with the original 3D DSA to evaluate the clinical usefulness and accuracy of the model. Figure 1 demonstrates a schematic overview.

## Methods

### Study design

This single-center retrospective observational study was ethically approved by the institutional review board. This retrospective study adhered to the 2013 Declaration of Helsinki. The institutional review board waived the requirement for informed consent due to the retrospective nature of the study. The data was processed without personal data. A defacing algorithm was not used (in the sense of true anonymization). All methods were performed in accordance with relevant guidelines and regulations.

### Participant population

We searched our PACS database for patients with complete 3D DSAs between 01/01/2018 and 12/31/2022. We excluded 3D DSAs with severe movement artifacts. The remaining data sets were divided randomly into training, validation, and test sets (66:17:17). For better statistical assessment, we created two subgroups from the test data: 3D DSA with and without severe artifacts (e.g. movement, stents, coils, clips…).

A neuroradiologists screened and divided the test data sets into these two subgroups (subjective decision: “no, minimal and minor overall artifacts” versus “moderate, severe and very severe overall artifacts”).

### Angiographic suite and technical details

We performed all 3D angiographies on a Siemens Axiom Artis Cath/Angio System (Siemens Healthineers AG, Werner-von-Siemens-Str. 1, D-80333 Munich, Germany) with the following parameters: 70 kV, matrix size of 512 × 512, voxel size of approx. 0.28 mm^3^.

Calculations were performed with edge-enhancing or Hounsfield-optimizing kernels (“Sub Medium EE Auto Mo” and “Nat Fill Medium HU Auto”, further parameter for both reconstructions: “image characteristics: auto”).

### Pre-processing

We optimized the contrast and the field of view of the input data (DICOM).

### Image-to-image translation with conditional generative adversarial networks (cGAN)

The pix2pix [6] translation was developed using this method to reconstruct objects from edge maps, synthesize photos, and to colorize images. We adapted the cGAN-framework published by Choi et al. [7] (https://github.com/jwc-rad/pix2pix3D-CT) to simulate subtraction images.

### Calculation of virtual subtraction images

We trained the cGAN based on pix2pix on 3D-DSA data with the aim to directly generate the subtraction series (sub) from the contrast-enhanced series (fill) using domain transfer without prior segmentation. The internal image shape was 128 × 128 × 128 with a grid interval of 64 × 64 × 64. Training was carried out with a batch size of 3 over 10 epochs. Other parameters include Adam (optimizer), a dropout rate of 0.2, and a learning rate scheduling of 0.00018 to 0.1. When training the discriminator, a random shift (with a 10% probability), a random vertical flip (30%) and additional noise (10%) were added to the input data. The programming language used was Python3 Version 3.9.18. The network training took place on a PC equipped with an Intel Core i7-11700, 64GB RAM and an NVIDIA GeForce RTX 3090 with 24GB GPU memory.

The adapted code of the software is available under github (https://github.com/University-Clinic-of-Neuroradiology/pix2pix3d-ct).

### Automated comparison of image equality

We automatically evaluated the image quality (or better image accuracy or equality) using Structural Similarity Index Measure (SSIM), Normalize Mean Square Error (NMSE) and Peak Signal to Noise Ratio (PSNR) [8].

### Manual comparison of image quality

We programmed an application to compare both sequences (3D DSA and virtual 3D DSA). With this software, five blinded raters (SM, RS, EK, SZ, ED) with more than five years’ experience each in neuroradiology evaluated the test set containing both scans with and without artifacts. Additionally, the contrast-enhanced non-subtracted series were provided to the raters (to clarify possible contradictions).

All raters had to complete a questionnaire for each case containing the following parameters, which was to be ranked on a Likert scale from 0 to 5:

- Overall image quality (0 – very poor; 1 – poor; 2 – fair; 3 – good; 4 – very good; 5 - excellent).
- Severity of artifacts caused by (1) metal implants or (2) movement/bone (0 – no artifacts; 1 – minimal artifacts; 2 – minor artifacts; 3 – moderate artifacts; 4 – severe artifacts; 5 – very severe artifacts).
- Quality of images for (1) small, (2) middle and (3) large vessels (0 – very poor; 1 – poor; 2 – fair; 3 – good; 4 – very good; 5 - excellent).

### Significance test

We tested the significant differences of groups with and without artifacts for SSIM, NMSE, PSNR using the Mann-Whitney-U-Test.

Difference between real and calculated DSA was tested using the Wilcoxon signed-rank test for overall image quality, metal artifact, movement/bone artifacts, small vessel, middle vessels and large vessels. We did not use a t-Test since parameters were not normally distributed.

We calculated a sample size of at least 64 for each of our test data sets (Confidence-Level 90%, margin of error 10%), following the instructions of Jones et al. [9].

### Statistical analysis

We used Python3 (Version 3.9.18, matPlotLib) for statistical programming and histogram / image construction. We calculated intraclass correlation coefficient (ICC, 3, k) using Python3 and the libraries “pandas” and “pingouin”. Interpretation of results was done following Koo and Li [10].

## Results

### Participants

**Fig. 2:**
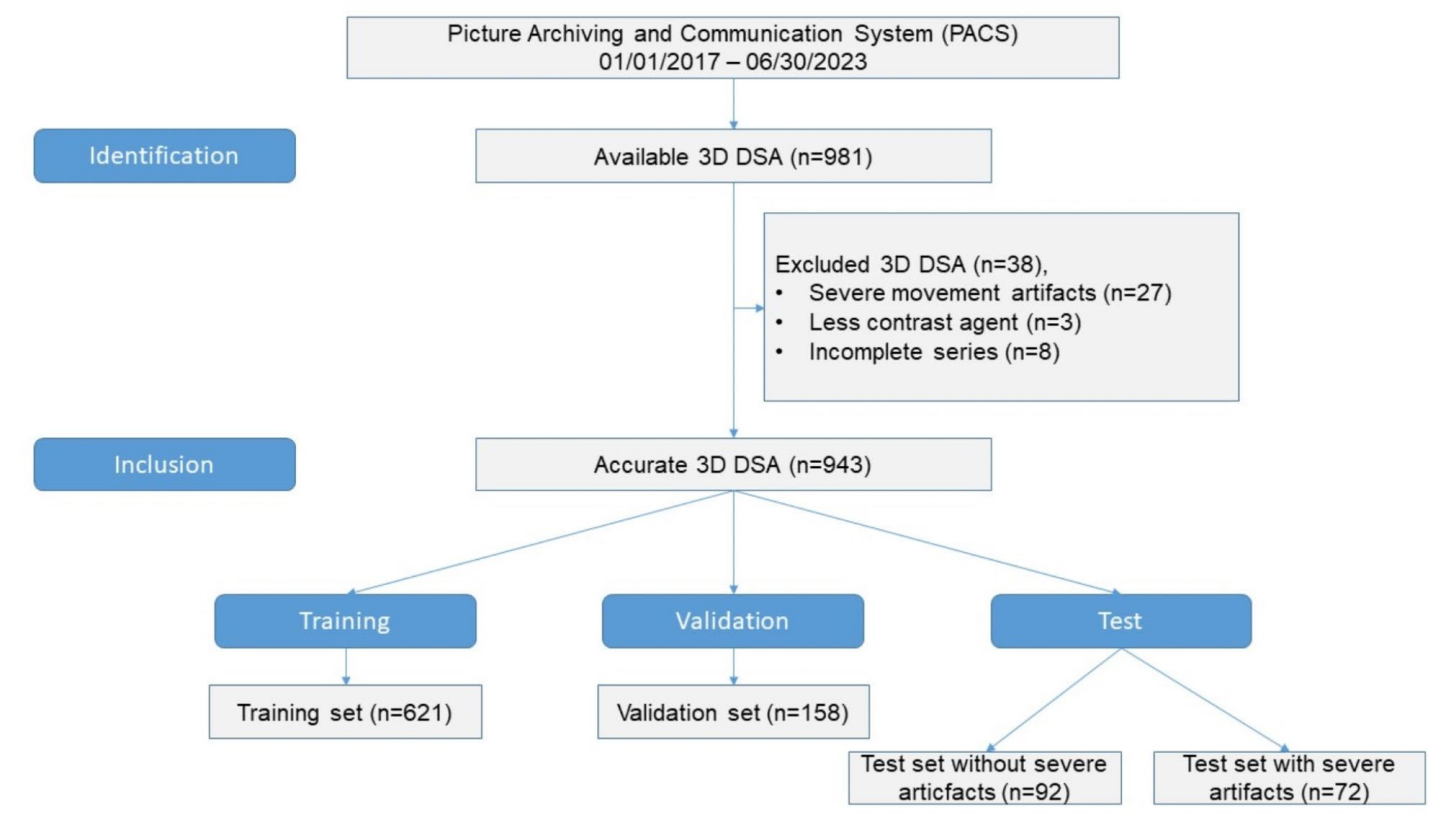
Included data sets

Our PACS search revealed 981 available 3D DSA (680 patients) between 01/01/2017 and 12/31/2022. We excluded 38 3D DSAs with a final count of 943 scans from 654 patients. Figure 2 shows a detailed flow chart of inclusion criteria. Mean patients age was 56.8 ± 13.2 (mean ± standard deviation) years. Indications for the angiographies and basic findings are listed in Table 1.

**Table 1:** Details of included 3D subtraction angiographies (*n* = 621, 158, 164)

	Training	Validation	Test without severe artifacts	Test with severe artifacts
**3D rotational DSA**	621	158	92	72
**Subarachnoid hemorrhage**	245 (39%)	86 (54%)	83 (90%)	15 (21%)
**Perimesencephalic subarachnoid hemorrhage**	37 (6%)	5 (3%)	2 (2%)	3 (4%)
**Aneurysm**	554 (89%)	146 (92%)	82 (89%)	61 (85%)
**Dural arteriovenous fistula**	2 (< 1%)	0 (0%)	0 (0%)	0 (0%)
**Arteriovenous malformation**	25 (4%)	6 (4%)	3 (3%)	7 (10%)
**Stent / Flow Diverter**	131 (21%)	53 (33%)	37 (40%)	14 (19%)
**Coils**	281 (45%)	99 (63%)	51 (55%)	29 (40%)
**Aneurysm clips**	193 (31%)	33 (21%)	12 (13%)	18 (25%)
**Ventricular shunt catheter**	31 (5%)	7 (4%)	3 (3%)	2 (3%)
**Other**	15 (2%)	4 (3%)	4 (4%)	3 (4%)

### Network training and calculation of virtual subtraction images

We trained and validated the cGAN with 779 data sets, 621 for training,158 for validation, and 164 for testing (split 66%:17%:17%). Tests were carried out using two different data sets: (A) with 92 cases with fewer artifacts (Data_woA_), and (B) with 72 cases with severe artifacts (Data_wA_).

### Automated comparison of image quality

**Fig. 3:**
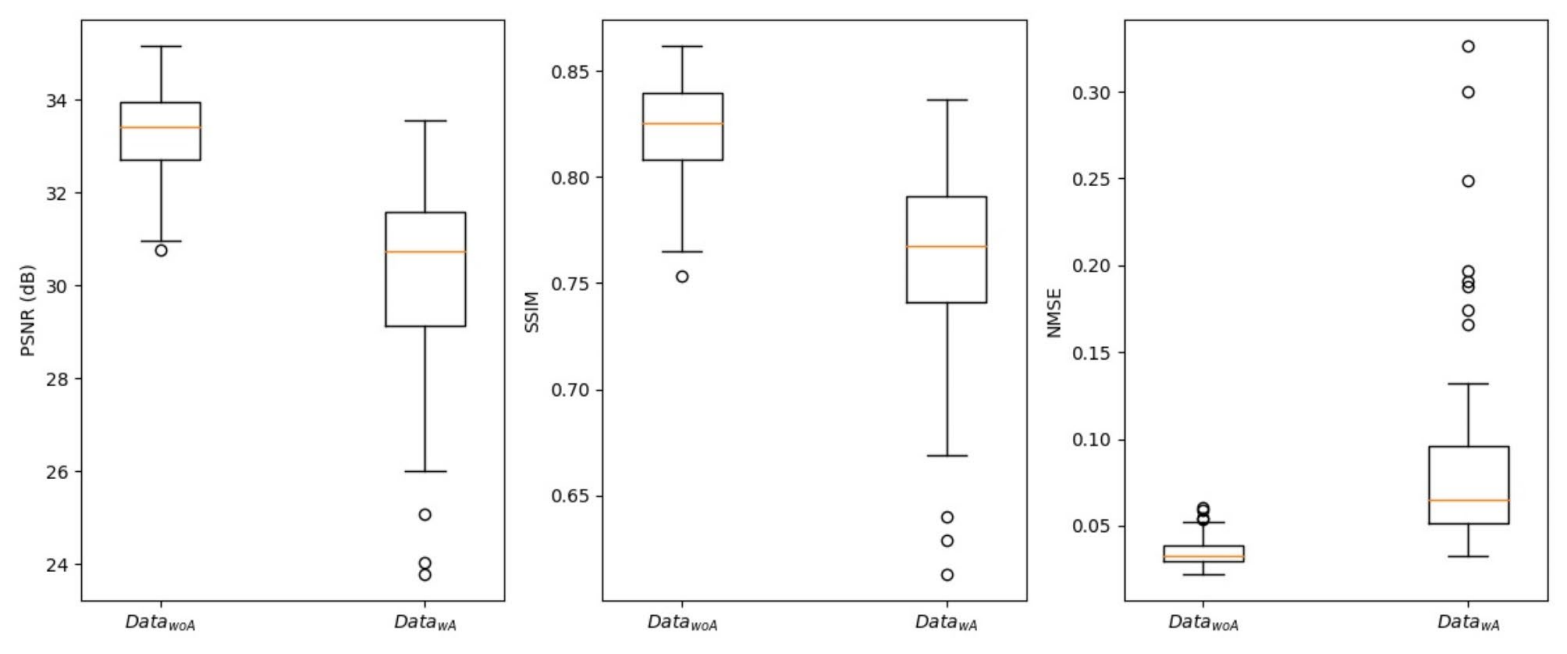
Results of PSNR, SSIM and NMSE from both test data sets Data_woA_ and Data_wA_ (calculated using virtual and real 3D subtraction angiographies; box plot: yellow line : median; box : IQR = Q3-Q1; whiskers_low = Q1–1.5 IQR; whiskers_high = Q3 + 1.5 IQR). **Legend**: PSNR - peak signal-to-noise ratio in dB; SSIM - structural similarity index measure (interval from − 1 to + 1); NMSE - normalized mean square error (interval from 0 to infinity); IQR - interquartile range

In our case, we used the PSNR, SSIM and NMSE metrics to quantify how good the images generated by the GAN were compared to the original subtraction. The results of the evaluation are depicted in Fig. 3.

The PSNR shows higher values for the Data_woA_ than for the Data_wA_, where higher values indicate better image quality. This indicates that the GAN in the data set without artifacts is closer to the original than the GAN in the data set with artifacts.

This is also apparent when looking at the SSIM results. The SSIM value is higher for Data_woA_ than for Data_wA_. The GAN reduces the movement/bone artifacts, which leads to a decrease in the similarity between the GAN image and the original subtraction image.

The NMSE results also support the previous findings. By avoiding the artifacts, the quadratic deviation between GAN and subtraction image increases, which leads to higher NMSE values for Data_wA_.

### Manual comparison of image quality

Manual comparison of image quality demonstrates lower bone artifacts for the generated 3D DSA compared to the original one. The algorithm visualized large vessels more homogeneously in data sets without artifacts.

Furthermore, we found no significant differences in the subjective image quality.

**Fig. 4:**
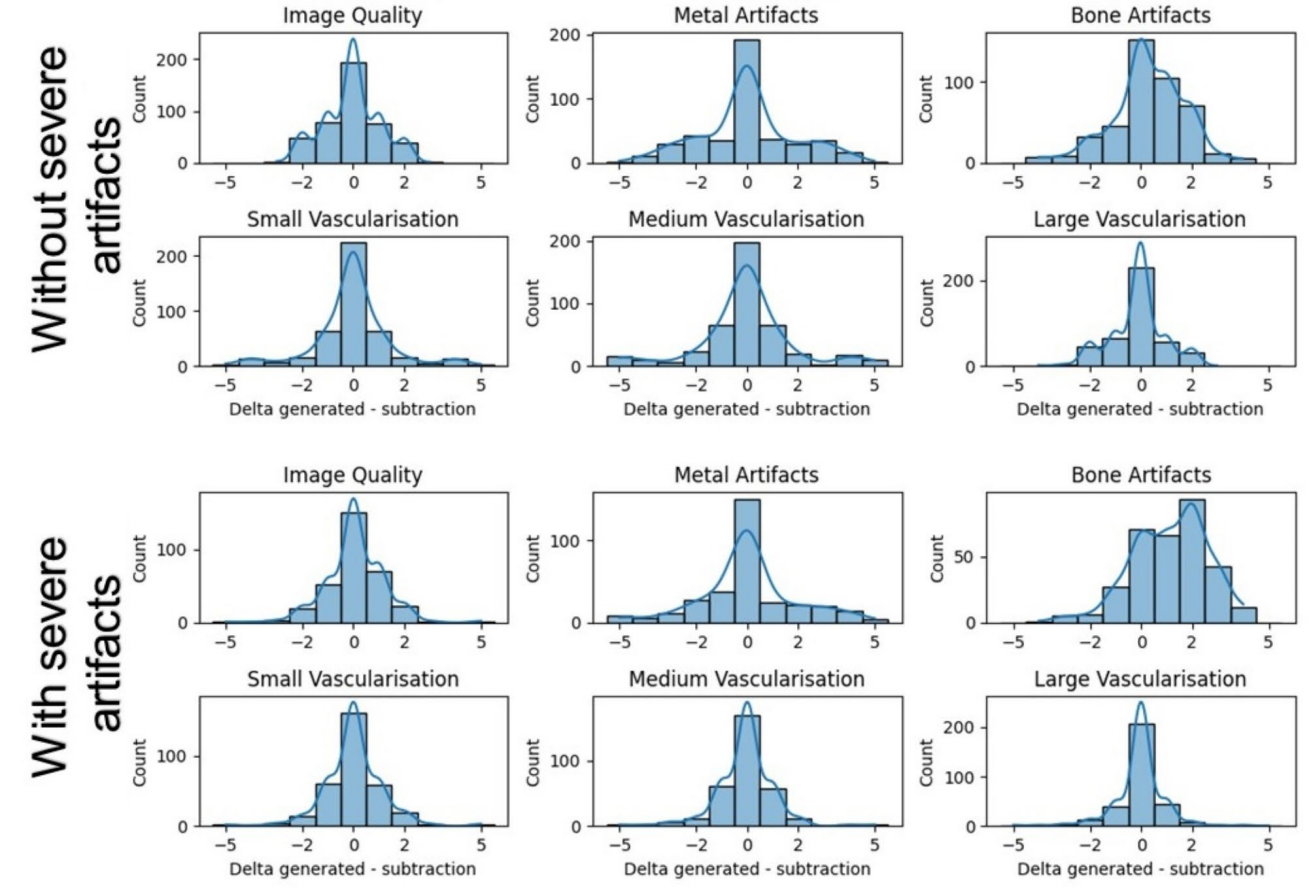
Delta (results of real 3D DSA minus virtual 3D DSA) of evaluation for the patients without (top) and with (bottom) severe artifacts caused. **Legend**: All parameters were rated at a Likert scale from 0 to 5. A high number suggests good quality for the parameters image quality, small, medium and large vessel. While for artifacts a low rating represents desirable quality. If the virtual DSA is rated better than the real one, negative numbers are possible

Figure 4 demonstrates differences of rated quality scores of virtual and real 3D DSA.

### Significance test

Mann-Whitney-U-Test revealed the following p-values for Data_woA_ versus Data_wA_:

*p* (SSIM) < 0.00001, *p* (NMSE) < 0.00001, *p* (PSNR) < 0.00001.

Table 2 lists the results of the comparison / significance tests of real 3D DSA and cGAN-generated 3D DSA.

**Table 2:** Results of the significance tests (Wilcoxon signed-rank test) of real 3D DSA vs. cGAN-generated 3D DSA

	Data_woA_	Data_wA_
**Overall image quality**	*p* = 0.301	*p* = 0.28
**Metal artifacts**	*p* = 0.791	*p* = 0.553
**Movement / bone artifacts**	*p* < **0.00001**	*p* < **0.00001**
**Small vessels**	*p* = 0.6	*p* = 0.727
**Middle vessels**	*p* = 0.48	*p* = 0.298
**Large vessels**	*p* = **0.04**	*p* = 0.408

**Legend**: bold – significant at a significance level of 5%

In the three group comparisons where a significant difference occurred, simulated 3D DSA showed better results (less movement/bone_artifacts and better visualization of larger vessels in angiographies without severe artifacts).

### Intraclass correlation

Intraclass correlation was between a moderate and good level (0.69–0.88) for all ranked parameters except for bone artifact which revealed only a poor reliability (0.29–0.48). Table 3 shows detailed intraclass correlation coefficients ICC(3, k).

**Table 3:** Intraclass correlation coefficients of five raters; two-way mixed, average measures, consistency ICC (3, k); mean [confidence interval]

	With artifacts	Without artifacts
**Image quality**	0.81[0.73; 0.87]	0.67[0.54; 0.77]
**Metal artifacts**	0.81[0.72; 0.87]	0.83[0.77; 0.88]
**Bone artifacts**	0.29[-0.03; 0.53]	0.48[0.28; 0.64]
**Small vessels**	0.75[0.64; 0.83]	0.88[0.83; 0.91]
**Medium vessels**	0.84[0.76; 0.89]	0.85[0.8; 0.9]
**Larger vessels**	0.79[0.69; 0.86]	0.70[0.58; 0.79]

**Legend**: ICC < 0.5 poor, between 0.5 and 0.75 moderate, between 0.75 and 0.9 good reliability

### Artifacts

**Fig. 5:**
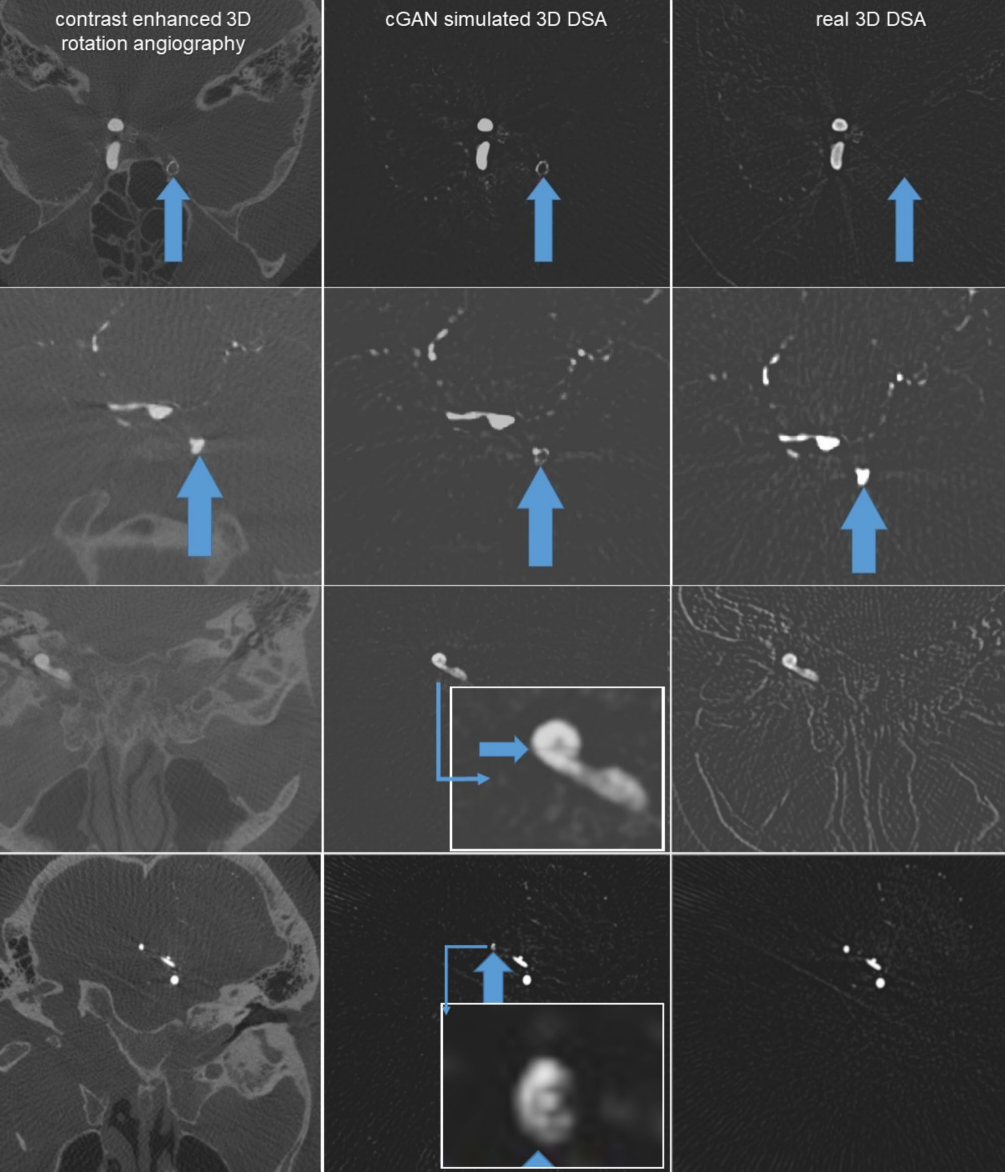
Artifacts: First row: A contralateral, non-contrasted intracranial stent (blue arrows). Since there were not enough cases of this in the training database, the generative AI incorrectly interpreted it as a “vessel”. Second row: the generative AI incorrectly interpreted a “vessel” as a “stent”. Third row: Grid composition artifact. Fourth row: unclear “stick figure artifact”, most likely due to grid composition error

We found many artifacts in the area of extinction around metal implants in both angiographies, but only a few (*n* = 7) cGAN created artifacts without severe metal extinction in the virtual images. Most of these were based on smaller bone or metal artifacts without sufficient correlation in the training data sets. Figure 5 demonstrates two examples of misinterpreted stents.

## Discussion

In our study of 943 rotational subtraction angiographies, we showed that generative AI can successfully simulate 3D DSA in a wide range of vascular diseases, and that this method can be used after endovascular therapy with metal artifacts. Since we did not implement the commonly used standard convolutional neuronal networks (CNNs), but rather generator and discriminator networks (based on CNNs as well), we increased the data volume to stabilize the training (to better estimate Nash’s equilibrium) [11, 12]. The mode collapse phenomena is reduced in cGANs compared with GANs [13]. Although the amount of data in cGAN does not necessarily have to be higher than in conventional CNNs, the number of training and test data sets in our study is significantly higher than previously published studies [14–17]. The simulated subtraction angiographies revealed no significant differences to the real DSAs in terms of quality and showed fewer artifacts overall. This allows us to reduce the radiation exposure by approximately 50% with the same image quality. In a few individual cases, there were major errors caused by severe coil artifacts, but they were generally obvious.

We found a better and more homogenous contrast visualization in large vessels since the algorithm smooths contrast agent flow phenomena.

In 2008, 3D rotational angiography was introduced as the new gold standard in the detection of intracranial aneurysms, with a larger sensitivity for smaller aneurysms (≤ 3 mm) than conventional 2D DSA [18]. Another study from Wong et al. confirmed the results and showed that up to 10% (3/31) of small aneurysms (≤ 5 mm) can be missed due to vascular overlay using 2-DSA, and therefore 3D rotational DSA is essential for better diagnostics [19]. A study by Duffis et al. emphasized that conventional 2D DSA itself offers only minor advantages over computed tomography angiography (CTA) in the detection of stenosis and clinical decision making [20].

The bottom line is that 3D rotational DSA is preferable to other methods for detecting vascular anomalies, stenoses or aneurysms due to its high resolution.

In 2018 Montoya et al. developed an algorithm based on CNNs to create a 3D cerebral angiogram from a single contrast-enhanced C-arm cone beam CT [14]. After training (*n* = 35), validation (*n* = 8) and testing (*n* = 62), the resulting “deep learning angiography” reduced motion and bone artifacts and enabled a 3D angiogram without native mask acquisition, and therefore reduced the radiation dose by approximately 50% [14].

In 2019, Lang et al. [15] were able to confirm these results in patients without pathological findings using CNNs as well (training set *n* = 98; test set *n* = 15). In another study from Lang et al. (training set *n* = 98), such artificial intelligence-based angiographies were successfully used for pre-interventional visualization of aneurysms (*n* = 10), dural arteriovenous fistulas (*n* = 10) and arteriovenous malformations (*n* = 10) [16]. Additionally, Lang et al. [17] reported initial positive experiences for the grading of intracranial artery stenoses using CNNs (training set *n* = 98; test set with stenoses *n* = 10). The applicability of this CNN architecture and dataset to 3D DSA micro imaging (training set *n* = 98, test set *n* = 20) has been demonstrated by Ishikawa et al. in a Japanese study [21].

### Limitations

The retrospective nature of the study represents a major limitation. True blinding of the raters was difficult due to the more uniform contrast agent behavior in large vessels and significantly fewer bone artifacts. We achieved acceptable reliabilities in the quality ratings with the exception of bone artifacts, which seemed to be more subjective.

We recorded and evaluated objective and subjective criteria for the calculated images. However, the diagnostic value of a 3D subtraction angiography depends on the corresponding diagnostic question. Therefore, it could not be determined with certainty. For this purpose, larger prospective studies with specific questions are necessary.

Whether we will be able to forego real 3D DSA for certain questions in the future must be clarified in further studies. In the rare cases where cGAN generated artifacts occurred, the exact reasons for this could not be clarified due to the non-transparent learning mechanism. The unchanged quality with regards to the metal artefacts is due to the diversity of metallic structures and the associated lack of availability in the training data sets. An expansion of the training data sets and further modifications to cGAN could improve results.

Another limitation is the use of two different kernels (non-uniform post-processing) for subtraction and contrast-enhanced scans, which could introduce a bias. The cGAN additionally learned to transform the “soft” kernel to an edge-enhancing kernel. The reconstruction option “image characteristics: auto” depends on the ratio of voxel size to detector pixel size. It should be constant, so no additional bias is to be expected here.

In many cases, non-contrast-enhanced 3D DSA will be indispensable in the future. In particular, precise imaging of the structure and wall apposition of flow diverters and stents is not possible without the information from native imaging. This also includes the planning of suitable projections in aneurysm therapy, whereby overlap with metal implants should be avoided.

Nevertheless, the method presented here is well suited to reduce the overall radiation exposure by improving the initial angiographic assessment or as a control before aneurysm treatment using metallic implants.

### Outlook

In 2021, Choi et al. created synthetic contrast-enhanced chest computed topographies’ from non-enhanced chest CTs using GANs (training set *n* = 25, validation set *n* = 25, test set *n* = 12) [7]. This technique enabled a higher detection rate of lymph nodes using simulated images compared to the native scan alone. Theoretically, such a procedure could also increase the detection of cerebral aneurysms in a native CT scan. However, the truth-value of the calculated images could potentially decrease significantly and the AI could simply “invent vascular structures” in cases with poor image quality.

Other promising approaches for algorithms using AI are the overlying of two volumes in complex vascular pathologies and / or with supply from multiple vascular territories [22].

Su et al. [23] introduced a CNN-based approach using 2D DSAs before and after interventions (“autoTICI score”) for the automated evaluation of mechanical thrombectomies.

A multi-network approach might potentially reduce metal artefacts, for example. Another network could be used to determine whether metallic structures are present and then, if necessary, a pipeline dedicated to metal artefacts could be created. The inclusion of additional data sets in the training process could also improve results.

## Conclusion

The application of generative AI to create synthetic 3D DSA allows for an excellent visualization of intracranial vessels with a lower radiation dose. However, there are still small snags that need to be eliminated with proper training data, especially in the cases where there are large aneurysms, stents and severe coil artifacts. After further developments, should the bugs and artifacts be successfully eliminated, the technology might one day be used in everyday clinical practice.

## Data Availability

The datasets used and/or analyzed during the current study available from the corresponding author on reasonable request.Python code is partially available from the corresponding author on reasonable request.

## Abbreviations

cGAN: conditional generative adversarial networks
CNNS: Convolutional neuronal networks
CT: Computed tomography
CTA: Computed tomography angiography
DSA: Digital subtraction angiography
IQR: Interquartile range
MRI: Magnetic resonance imaging
MR: Magnetic resonance
SD: Standard deviation
